# Influence of Combustion Modifiers on the Cure Kinetics of Glycidyl Azide Polymer Based Propellant-Evaluated through Rheo-Kinetic Approach

**DOI:** 10.3390/polym11040637

**Published:** 2019-04-08

**Authors:** Liming He, Jun Zhou, Sulan Dai, Zhongliang Ma

**Affiliations:** School of Environment and Safety Engineering, North University of China, Taiyuan 030051, China; 18334788227@163.com (J.Z.); dsl1996@163.com (S.D.)

**Keywords:** GAP spherical propellant, combustion modifier, rheological method, cure kinetics

## Abstract

To investigate the influence of combustion modifiers on the curing of glycidyl azide polymer spherical propellants (GAPSPs), the curing process of the GAPSPs was explored using an isothermal rheological measurement method. The parameters of cure kinetics were solved to further establish a kinetic model for the curing reaction of GAPSPs. The results showed that the curing process of GAPSPs under isothermal conditions conformed to the Kamal and LSK (Lu–Shim–Kim) models. The model data indicated significant agreement with the experimental data. The influence of four kinds of combustion performance modifiers on the curing process was explored and the results demonstrated that lead phthalate had a catalytic effect on the curing reaction of GAPSPs, whilst oxides of lead and copper, and copper adipate had no influence on the curing reaction.

## 1. Introduction

Glycidyl azide polymer (GAP), as a novel azeotropic binder, has been widely used in solid propellants and paste propellants [1,2,3,4,5]. GAP propellant has the advantages of high energy, high density, and a low characteristic signal; however, its mechanical properties are needed to be improved [6,7]. Michael [8] mixed GAP with nitrocellulose (NC) and introduced it into the propellant to improve the energy and mechanical properties of the propellant. Luo Yunjun et al. [9,10,11] combined GAP with NC to obtain novel GAP modified spherical propellants (GAPSPs), which were applied to a cross-linked modified double-base propellant and achieved good results. Since the curing of the propellant has an important effect on its performance, it is very necessary to study the curing kinetics of the GAPSPs.

Common methods for studying the curing process include differential scanning calorimetry (DSC) [12], infrared spectroscopy (FTIR) [13], etc. The curing of GAPSPs is the reaction of hydroxyl groups on the molecular chain of NC and GAP with isocyanate. Since NC is a solid, the curing of GAPSPs and isocyanate belongs to the heterogeneous curing reaction system. There are absorption, dissolution, and diffusion processes in the initial stage of the reaction, and a diffusion-based process in the late stage of the reaction. The thermal analysis method will produce a larger error. Recently, domestic and foreign scholars have applied rheometers to study reaction kinetics and they achieved good results [14,15,16,17]. The advantage of the rheological measurement method is that it can more intuitively reflect the modulus change of the system during the curing process, and it can intuitively describe the state change of the curing system, which has an important guiding role for the actual production process.

We used a rheological method to investigate the curing kinetics of GAPSPs in other studies [18,19,20,21]. In this paper, we focused on the curing mechanisms of GAPSPs. Additionally, the influences of combustion modifiers on GAPSPs were explored to provide a theoretical basis for the application of GAPSPs.

## 2. Materials and Methods

### 2.1. Samples

GAPSPs were prepared with a GAP mass fraction of 30% and a NC mass fraction of 70%, and GAP were supplied by LiMing Chemical Industry Institution in Luoyang, China. Isophorone diisocyanate (IPDI) was purchased from Mingda New Material Co., Ltd. in Jinan, China, and used as the curing agent. Dibutyltin dilaurate (DBTDL) was supplied by Taizheng Chemical Company in Shanghai, China and was employed in the form of 5% solution in dibutyl phthalate as curing catalysts. The combustion modifiers used in the present study were lead oxide, lead phthalate, cupric oxide, and copper adipate, which were supplied by Shanxi Xingyuan Chemical Trading Co., Ltd. in Taiyuan, China.

### 2.2. Experimental Instruments and Conditions

An MCR 302 rheometer manufactured by Anton Par GmbH in Graz, Austria was used for the experiments.

There are two kinds of rheometer clamps: Cone plate and parallel plate. Given that the cone plate cannot be used for a suspension, as the original state of the test material is suspension, the parallel plate clamp is used. The maximum range of the parallel plate gap is 500 μm–0.1d (d is the clamp diameter 25 mm), and the gap should be about 10 of the maximum particle size of the material. The GAPSP particle size is approximately 100 μm, so the fixture gap is 1.0 mm.

The storage and loss moduli of the samples were measured using an amplitude scanning mode at the frequency of 1 rad. The strain amplitude was 1% and the strain was sustained within the linear viscoelasticity. The tests were conducted at 30, 40, 50, and 60 °C.

### 2.3. Test Methods and Curing Model

The curing process of GAPSPs and IPDI is considered as a comprehensive process involving chemical reactions and physical diffusion [22]. During the initial stages of the reaction, storage modulus rapidly increased and the rate decreased as reaction progressed and eventually reached a limiting value beyond which the increase in storage modulus was almost nil. The conversion rate of the reaction can be characterized using the ratio of the modulus increment at *t* to that during the whole curing reaction as described in Reference [23], which can be calculated using Formula (1).

(1) $$\alpha =\frac{G_{t}^{\prime }-G_{0}^{\prime }}{G_{\infty }^{\prime }-G_{0}^{\prime }}$$

where α refers to the conversion rate of the reaction, while $G_{t}^{\prime }$, $G_{0}^{\prime }$, and $G_{\infty }^{\prime }$ represent the storage moduli at *t*, and at the initial and final stages of the reaction, respectively. The denominator denotes the contribution of the curing reaction to the storage modulus $G^{\prime }$ during the whole reaction, while the numerator refers to the contribution of the curing reaction before the time *t* to the storage modulus $G^{\prime }$.

The basic rate equation can be expressed as in Formula (2).

(2) $$\frac{d\alpha }{dt}=k\left(T\right)f\left(\alpha \right)$$

where $\frac{d\alpha }{dt}$ refers to the curing rate and k(T) denotes the influence of the temperature on the rate, which is generally expressed using the Arrhenius equation. Moreover, f(α) is a function on the extent of reaction.

The curing reaction is complex and involves multiple elementary reactions. The common mechanism functions include an *n*-order reaction model (Formula (3)), a Sestak–Berggren model (Formula (4)), and a Kamal model (Formula (5)) [24,25,26].

(3) $$\frac{d\alpha }{dt}=k{\left(1-\alpha \right)}^{n}$$

(4) $$\frac{d\alpha }{dt}=k{\left(1-\alpha \right)}^{n}\alpha^{m}$$

(5) $$\frac{d\alpha }{dt}=(k_{1}+k_{2}\alpha^{m}){\left(1-a\right)}^{n}$$

where, *m* and *n* both represent reaction orders.

When investigating the curing reaction of polymers, Lu et al. [27,28] suggested that the formation and growth of micro-gels in the curing process is similar to that of grains during crystallization. Based on the Avrami phase transition theory, the macro-kinetics model (Lu–Shim–Kim, referred to as the LSK curing model) for the curing reaction of thermosetting resin was deduced, as shown in Formula (6).

(6) $$\frac{d\alpha }{dt}=(k_{1}+k_{2}\alpha^{p}){\left(\alpha_{ep}-a\right)}^{n}$$

The parameter $\alpha_{ep}$ denotes the final curing degree under the equilibrium state of curing on the isothermal condition (considering $\alpha_{ep}$ = 1). Therefore, Formula (6) can be written as

(7) $$\frac{d\alpha }{dt}=(k_{1}+k_{2}\alpha^{p}){\left(1-a\right)}^{n}$$

where, $k_{1}$ and $k_{2}$ all refer to the functions of temperature in which $k_{1}$ and $k_{2}$ separately represent the rates of nucleation and growth. Moreover, *p* and *n* separately represent the growth and formation mechanism of the micro-gels.

The Kamal model (Formula (5)) and the LSK model (Formula (7)) have the exact same equation form, except that the parameters of the LSK model have a clear physical meaning. Consequently, the LSK model with clear physical meanings for each parameter can be considered as the valid phenomenological model to study the curing process.

The maximum reaction rate of the n-order model appears at α = 0, while the maximum reaction rate of the Kamal and LSK cure models appears in the middle of the reaction. Based on these characteristics, the type of models for the curing reaction can be determined.

## 3. Results and Discussion

### 3.1. The Apparent Activation Energy of the GAPSP Curing Reaction

**Figure 1:**
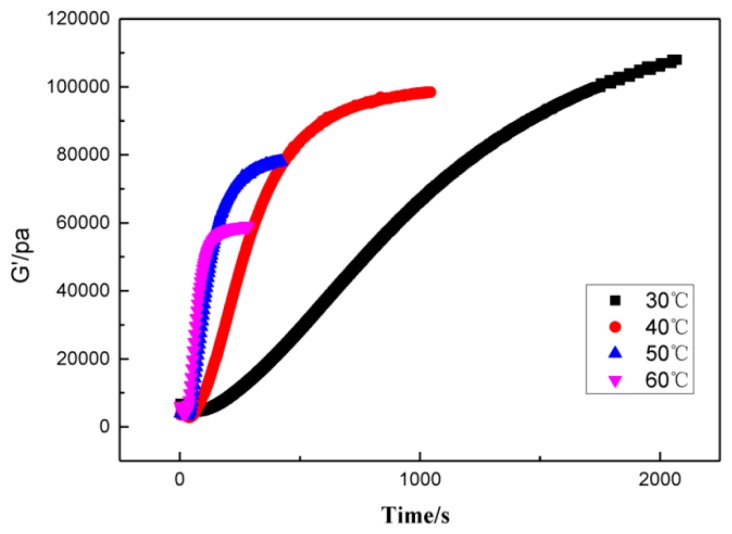
The change law of the storage modulus of slurries of GAPSPs with time.

Using a curing kinetics method based on isothermal conditions, the curing process of GAPSPs was investigated. By carrying out amplitude scanning at constant temperatures of 30, 40, 50, and 60°C, the change law of the storage modulus of slurries at different temperatures with time is shown in Figure 1.

**Figure 2:**
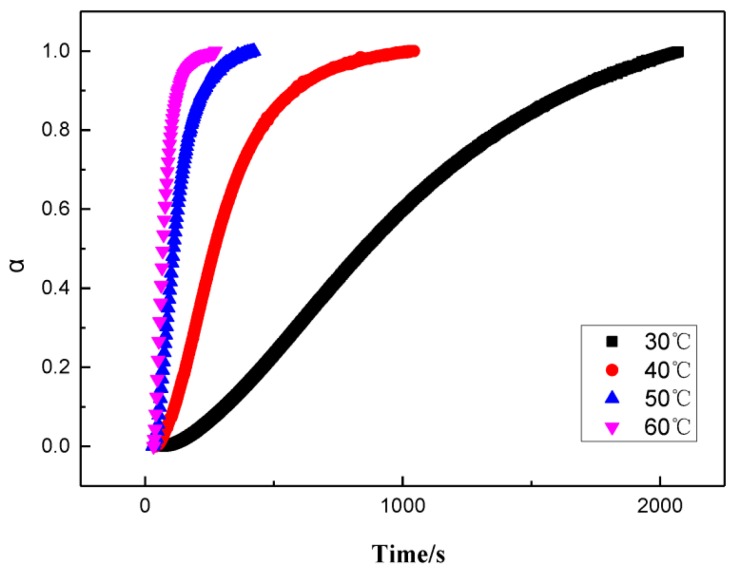
The relationship curve between the curing degree of the GAPSPs and time.

**Figure 3:**
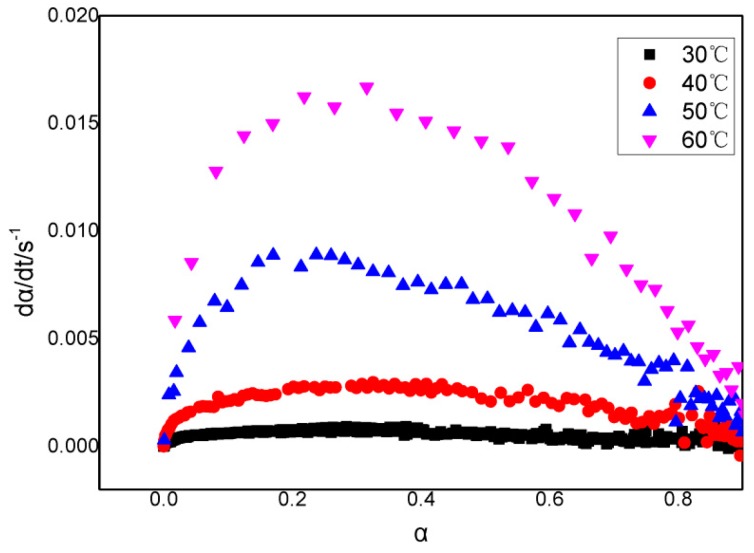
The relationship curve between the curing rate and the curing degree of GAPSPs.

From Figure 1, it could be seen that the storage modulus of slurries increased with the growing time. The lower the temperature, the slower the rate of increase of the storage modulus and eventually the larger the modulus. This could be explained as temperature increases the polymer chain tends to be more flexible which causes modulus reduction. Therefore, the maximum modulus was large. The storage modulus was transformed into a curing degree using Formula (1). Then, the relationship between the curing degree, time, and the curing rate can be acquired, as shown in Figure 2 and Figure 3, respectively.

As shown in Figure 3, at each temperature, the rate of reaction records a maximum when α is around 0.3 and the value of the maximum rate increases with temperature.

Adopting Freidman approach [29,30,31,32], activation energy was calculated from the slopes of the lines obtained by plotting ln(da/dt) against 1/*T*. The Friedman equation is shown in Formula (8).

(8) $$\ln\left(\frac{da}{dt}\right)=\ln\left[Af\left(a\right)\right]-\frac{E_{a}}{RT}$$

**Figure 4:**
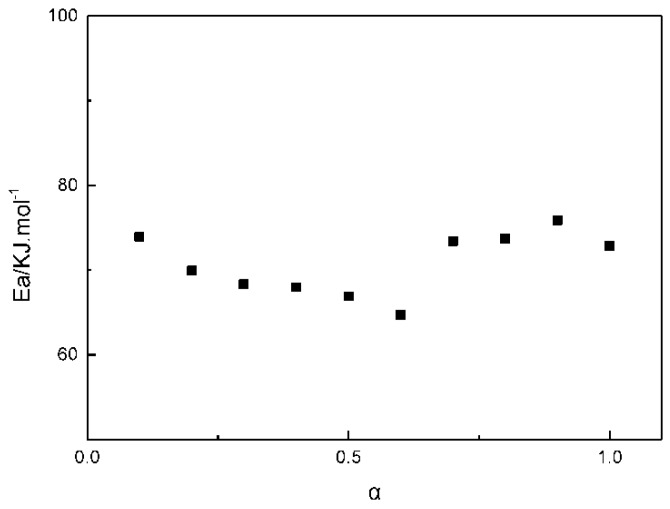
The change of the apparent activation energies during the curing reaction of GAPSPs with the conversion rate.

The calculated activation energy is shown in Figure 4.

From Figure 4, it could be seen that the activation energy in the whole curing process was in the range of 65–77 kJ/mol. The apparent activation energy of the GAPSP and IPDI reaction was greater than the apparent activation energy of the liquid GAP and IPDI reaction shown in the literature [33]. This was because the diffusion effect during the curing process was significantly increased, and the resistance of the system was also increased. At the initial stage of the curing reaction, the activation energy during the curing reaction of GAPSPs decreased slightly with the constant conduction of curing. This was because the initial stage of curing was primarily a kinetic control stage, where the content of hydroxyl groups in the system was high, and this could catalyze the curing reaction [34]. Overall, the activation energy is nearly constant for various values. This implies that mechanism of reaction does not get changed during the course of the reaction.

### 3.2. A model for the Curing Reaction of GAPSPs

**Figure 5:**
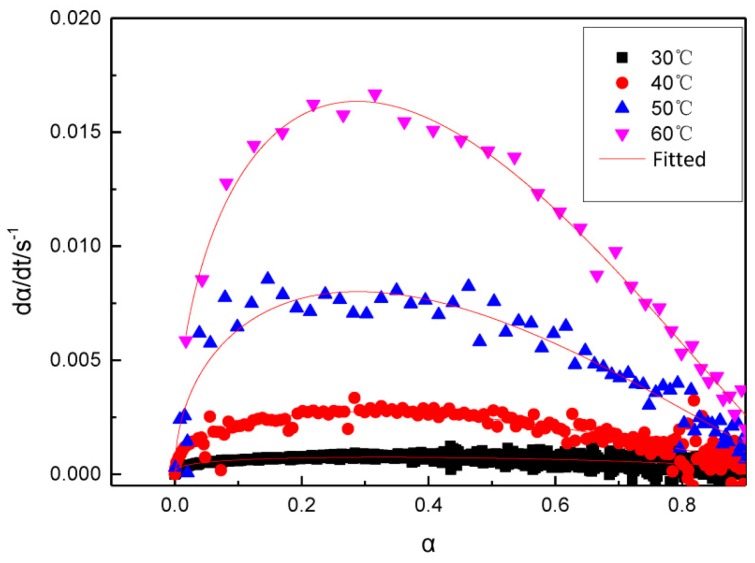
Comparison between the fitted data using the model and the experimental data.

Using the Kamal model and the LSK curing model, the curves for the curing processes of GAPSPs under isothermal conditions at four different temperatures were fitted, as shown in Figure 5.

The model parameter values at different temperatures were obtained from the fitting results and are listed in Table 1.

As shown in Figure 5, the Kamal model and the LSK curing model can fit the curing process of GAPSPs well, with the correlation coefficients being larger than 95%. The study found that the curing reaction of liquid GAP and IPDI was a second-order kinetic reaction [35], which was different to the curing mechanism of GAPSP. The reason was that, for the GAP curing reaction, the reactant GAP and the curing agent were both in the liquid phase, belonging to a homogeneous reaction. The reactant molecules had a large degree of freedom, and the reaction was carried out by mutual collision activation, where the concentration of the reactants was larger, and colliding with each other. The higher the probability, the larger the reaction rate. As the reaction progresses, the concentration of the reactants becomes lower and lower, and the reaction rate becomes smaller and smaller. Therefore, the GAP curing reaction mechanism function can be expressed by a second-order reaction mechanism function, and the maximum reaction rate appears at the beginning of the reaction.

This study indicated that the curing process of GAPSPs conformed to the Kamal and the LSK curing model which maximum reaction rate of system appears in the middle of the reaction. The reason was that, at the beginning of the reaction, the molecular chain grows in a linear and branched manner, and the molecular weight increases slowly. As the degree of reaction increases, the cross-linking point increases, the molecular weight increases rapidly, and the storage modulus increases rapidly. Therefore, the maximum reaction rate appears in the middle of the reaction.

### 3.3. GAPSPs Curing Kinetic Equation

According to Formula (7), the following formula can be acquired.

(9) $$\frac{d\alpha }{dt}=k_{1}{\left(1-\alpha \right)}^{n}+k_{2}\alpha^{p}{\left(1-a\right)}^{n}$$

where, $k_{1}$ and $k_{2}$ refer to the rate constants. Based on the Arrhenius equation, the following formula can be deduced:

(10) $$\frac{d\alpha }{dt}=A_{1}exp\left(-\frac{E_{\alpha ,1}}{RT}\right){\left(1-\alpha \right)}^{n}+A_{2}exp\left(-\frac{E_{\alpha ,2}}{RT}\right)\alpha^{p}{\left(1-\alpha \right)}^{n}$$

where, $E_{\alpha }$, *A*, and *R* represent the reaction activation energy, pre-exponential factor, and gas constant, respectively. Table 1 lists the k_1_, k_2_ values for curing at different temperatures. According to the Arrhenius equation, the diagram of ln*k*~1/*T* was a straight line. Based on the intercept and slope of the straight line, the activation energy $E_{\alpha }$ and the pre-exponential factor *A* could be acquired, while the values of the model parameters (*p* and *n*) were the averages at different temperatures [18,19]. By substituting various parameters into Formula (7), the kinetic equation for the curing reaction of GAPSPs can be established.

(11) $$\frac{d\alpha }{dt}=2.66\times 10^{9}exp\left(-\frac{7.72\times 10^{4}}{RT}\right){\left(1-\alpha \right)}^{1.1}+1\times 10^{15}exp\left(-\frac{1.03\times 10^{5}}{RT}\right)\alpha^{0.5}{\left(1-\alpha \right)}^{1.1}$$

**Figure 6:**
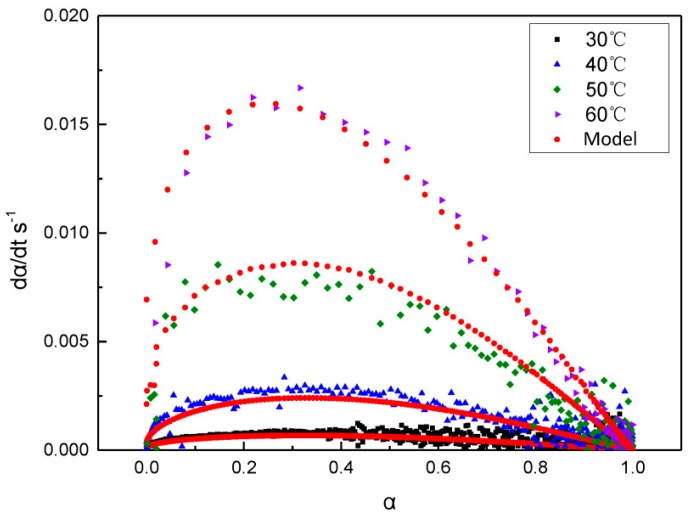
The curing rate (dα/dt)—conversion rate (*α*) curve of GAPSPs and its fitting curve.

A comparison of the curing rate curves for the reaction of GAPSPs at different temperatures and the fitting curves of the kinetic equation is shown in Figure 6. It can be seen that the fitting curves showed a good agreement with the rate curves, which implied that the kinetic equation for the curing reaction of GAPSPs could be used to describe the curing process of GAPSPs.

### 3.4. The Influence of Combustion Modifiers on the Curing of GAPSPs

**Figure 7:**
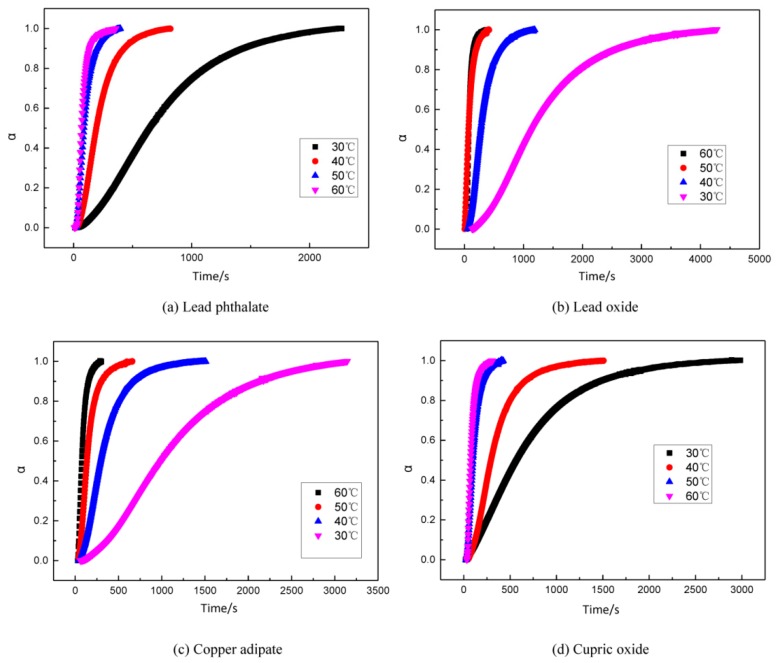
The change curve of storage modulus of the GAPSPs with time.

**Figure 8:**
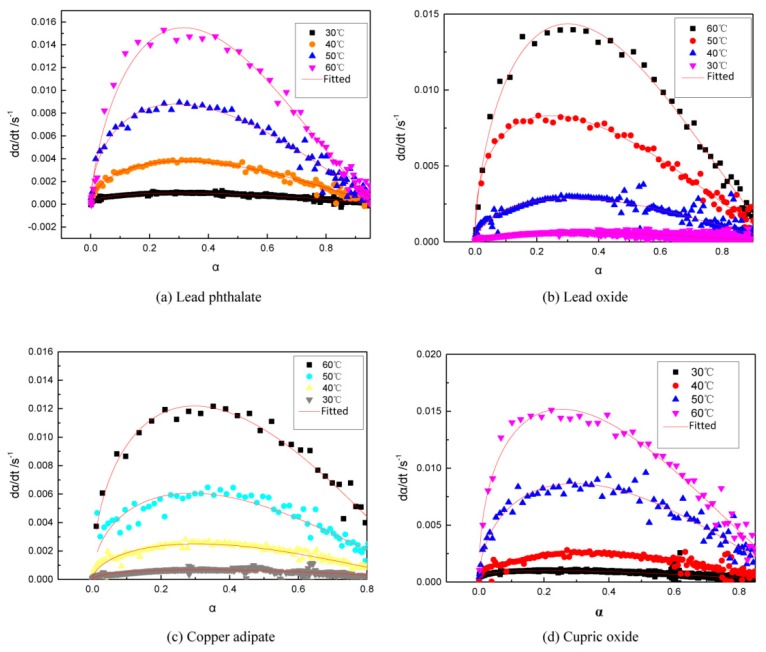
The conversion speed–conversion rate curve of GAPSPs and its fitted curve.

**Figure 9:**
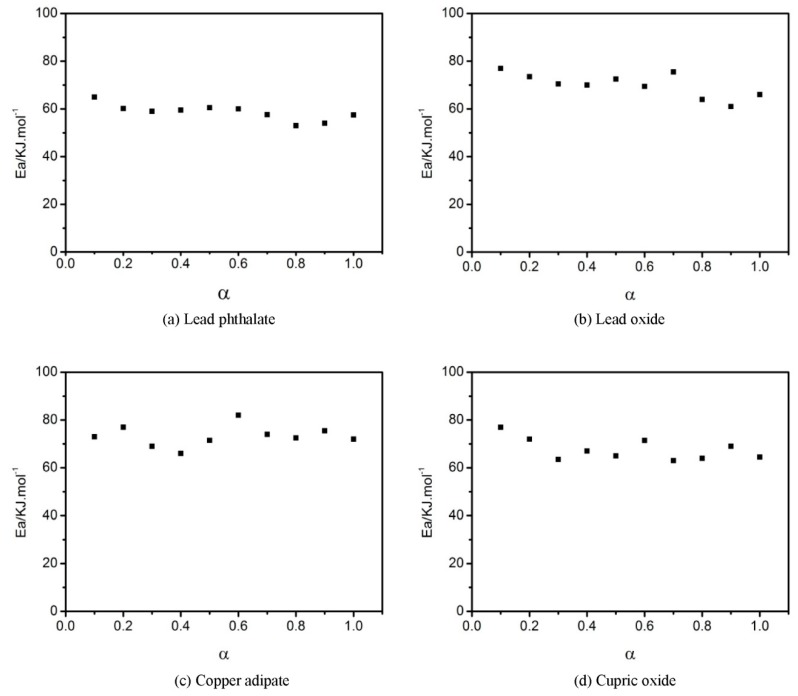
The relationship between the activation energy and conversion rate of GAPSPs.

Combustion modifiers in propellants generally are metal salts, which are expected to influence the curing reaction of the propellants. For example, some combustion modifiers react with curing agents so that the binder system fails to cure. Some combustion modifiers have an acceleration effect on the curing reaction, causing slurries to rapidly thicken which cannot be cast. It is necessary to investigate the influence of combustion modifiers on the curing reaction. Commonly used combustion modifiers in double-base and modified double-base propellants are a mixture of lead salt, copper salt, and carbon black. Using two types of lead salt (lead oxide and lead phthalate) and two kinds of copper salt (cupric oxide and copper adipate), their influences on the curing reaction of GAPSPs were investigated. The experimental results of curve fitting and activation energies during the curing reaction are separately displayed in Figure 7, Figure 8 and Figure 9.

Using the LSK curing model, the data obtained through the curing test after adding different combustion modifiers were fitted and the results are shown in Figure 8.

The activation energy during curing was calculated using the Friedman equation and the results are displayed in Figure 9.

From Figure 9, it can be seen that the activation energy of the system during the curing reaction was in the range of 53–65 kJ/mol after adding lead phthalate, which was lower compared to a range of 65–77 kJ/mol without adding the combustion modifiers. It revealed that lead phthalate showed a catalytic effect on the curing process of GAPSPs. After adding the three types of combustion modifiers (lead oxide, copper adipate, and cupric oxide) into the system, the activation energy during the curing reaction ranged from 65 to 80 kJ/mol, which approximated a range from 65–77 kJ/mol of activation energies from the system in the scenario without adding the combustion modifiers. These data implied that lead oxide, copper adipate, and cupric oxide had no catalytic effect on the curing process. The influence of different combustion modifiers on the curing reaction was closely related to the properties of metal cations in the catalysts. DBTDL is a commonly-used catalyst which is applied to catalyze the reaction between hydroxyl groups and isocyanate to further produce polyurethane (PU) [36]. The catalytic mechanism of DBTDL is due to hydroxylic hydrogen being activated based on the complex between tin ions and hydroxylic oxygen [37]. Both lead and tin belong to IV A-system elements in the periodic table and lead ions showed virtual orbits similar to tin ions.

## 4. Conclusions

Using a dynamic rheological method, the curing process and mechanism of GAPSPs were explored to further analyze the influences of curing and combustion modifiers on the curing reaction of GAPSPs. The following conclusions can be drawn from this study:

(1) The curing process of GAPSPs under isothermal conditions conformed to the Kamal model and the LSK model. The apparent activation energy of the modified GAP modified spherical drug is 65–77 kJ/mol, and the curing kinetic equation is

$$\frac{d\alpha }{dt}=2.66\times 10^{9}exp\left(-\frac{7.72\times 10^{4}}{RT}\right){\left(1-\alpha \right)}^{1.1}+1\times 10^{15}exp\left(-\frac{1.03\times 10^{5}}{RT}\right)\alpha^{0.5}{\left(1-\alpha \right)}^{1.1}$$

(2) The influences of four types of commonly used combustion modifiers on the curing reaction of GAPSPs were investigated. The results showed that lead phthalate showed a certain catalytic effect on the curing process, while lead oxide, cupric oxide, and copper adipate had minimal influence on the curing reaction.

## Figures and Tables

**Table 1:** The fitting model parameter values.

*T* (°C)	*k*_1_/*s*^−1^	*k*_2_/*s*^−1^	*p*	*n*	Correlation Coefficients
30	0.000245	0.00109	0.462	0.655	0.950
40	0.000102	0.0086	0.834	1.272	0.959
50	0.00163	0.0238	0.671	1.276	0.970
60	0.00214	0.0458	0.442	1.203	0.993
